# Age-specific cerebral haemodynamic effects of early blood pressure lowering after transient ischaemic attack and non-disabling stroke

**DOI:** 10.1177/23969873211039716

**Published:** 2021-09-04

**Authors:** Sara Mazzucco, Linxin Li, Iain J McGurgan, Maria A Tuna, Nicoletta Brunelli, Lucy E Binney, Peter M Rothwell, on behalf of the Oxford Vascular Study Phenotyped cohort

**Affiliations:** 1 Nuffield Department of Clinical Neurosciences, Wolfson Centre for Prevention of Stroke and Dementia, 6396 University of Oxford; 2 Campus Bio-Medico University of Rome, Rome, Italy

**Keywords:** Transient ischaemic attack, non-disabling stroke, blood pressure, age, transcranial Doppler

## Abstract

**Introduction:**

There is limited knowledge of the effects of blood pressure (BP) lowering on cerebral haemodynamics after transient ischaemic attack (TIA) and non-disabling stroke, particularly at older ages. We aimed to evaluate changes in transcranial Doppler (TCD) haemodynamic indices in patients undergoing early blood pressure lowering after TIA/non-disabling stroke, irrespective of age.

**Patients and methods:**

Among consecutive eligible patients attending a rapid-access clinic with suspected TIA/non-disabling stroke and no evidence of extra/intracranial stenosis, hypertensive ones underwent intensive BP-lowering guided by daily home telemetric blood pressure monitoring (HBPM). Clinic-based BP, HBPM, End-tidal CO_2_ and bilateral middle cerebral artery (MCA) velocity on TCD were compared in the acute setting versus one-month follow-up; changes were stratified by baseline hypertension (clinic-BP≥140/90) and by age (<65, 65–79 and ≥80).

**Results:**

In 697 patients with repeated TCD measures, mean/SD baseline systolic-BP (145.0/21.3 mmHg) was reduced by an average of 11.3/19.9 mmHg (*p* < 0.0001) at one-month (133.7/17.4 mmHg), driven by patients hypertensive at baseline (systolic-BP change = −19.0/19.2 mmHg, *p* < 0.001; vs −0.5/15.4, *p* = 0.62 in normotensives). Compared with baseline, a significant change was observed at one-month only in mean/SD MCA end diastolic velocity (EDV) (0.77/7.26 cm/s, *p* = 0.005) and in resistance index (RI) (−0.005/0.051, *p* = 0.016), driven by hypertensive patients (mean/SD EDV change: 1.145/6.96 cm/s *p* = 0.001, RI change −0.007/0.06, *p* = 0.014). Findings were similar at all ages (EDV change – p_trend_=0.357; RI change – p_trend_=0.225), including 117 patients aged ≥80. EDV and RI changes were largest in 100 patients with clinic systolic-BP decrease ≥30 mmHg (mean/SD EDV change = 2.49/7.47 cm/s, *p* = 0.001; RI change −0.024/0.063, *p* < 0.0001).

**Conclusion:**

There was no evidence of worsening of TCD haemodynamic indices associated with BP-lowering soon after TIA/non-disabling stroke, irrespective of age and degree of BP reduction. In fact, EDV increase and RI decrease observed after treatment of hypertensive patients suggest a decrease in distal vascular resistance.

## Introduction

Treatment of hypertension is safe and effective in elderly patients in primary prevention,^1–3^ with no evidence of cerebral hypoperfusion.^4^ Blood pressure (BP) lowering after stroke or transient ischaemic attack (TIA) also reduces the risk of recurrent stroke,^5,6^ but some uncertainties remain. Firstly, there is limited evidence on the effectiveness of BP-lowering in randomised trials in older (>80 years) patients with TIA/stroke.^6^ In most trials on antihypertensive treatment for secondary stroke prevention, including the PROGRESS trial,^5^ older patients were underrepresented. Secondly, there is uncertainty about how early antihypertensive treatment should be initiated. There is no evidence of benefit, and even possible harm, from acute initiation of treatment in major acute stroke.^7^ Although patients with TIA/non-disabling stroke are also often started on antihypertensive treatment in the acute setting,^8^ the effects of intensive blood pressure lowering on cerebral perfusion in these patients are uncertain, particularly at older ages. Small studies on TIA/stroke patients^9,10^ without carotid occlusive disease showed no evidence of worsening cerebral perfusion, but observation was limited to 2 weeks after initiating BP-lowering, and few older patients were studied. Reduced cerebral blood flow at older ages could place elderly patients at higher risk of cerebral hypoperfusion,^11^ particularly those with decreased diastolic flow and increased cerebrovascular resistances, which is associated with cognitive decline.^11,12^

Guidelines for management of hypertension in primary prevention set more cautious BP-thresholds as treatment goals in older patients,^13^ but guidelines for TIA/stroke make no comment on BP-lowering in older patients and do not address the timing of treatment.^7,8,14,15^ Uncertainty about age-specific thresholds and about timing might partly explain underprescription of antihypertensive medications after TIA/stroke,^16–18^ particularly if decisions are delayed, given that in-hospital prescription is known to be the strongest predictor of long-term adherence.^17^ High blood pressure in hypertensive subjects is associated with reduced cerebral blood flow,^19,20^ and with further decline with increasing age in longitudinal cohorts,^21,22^ but there is evidence of an increase in cerebral blood flow after intensive blood pressure lowering in older hypertensive subjects without history of stroke.^4^ In the absence of randomised trials comparing different BP-targets in elderly patients with TIA/non-disabling stroke, or of immediate versus delayed initiation of medication, data on the physiological effects of early BP-reduction might provide some support for clinical decision making. We hypothesised that intensive BP-lowering would not decrease transcranial Doppler (TCD) blood flow velocities, and in particular end-diastolic velocity, in older patients with recent TIA/stroke with no intra/extracranial carotid stenosis.

## Methods

We studied TCD parameters in a large population-based cohort attending a rapid-access clinic, undergoing early blood pressure lowering soon after TIA/non-disabling stroke, irrespective of age. The study was nested in the Oxford Vascular (OxVasc) Study^23^ (Supplementary Methods). From 1 November 2011, all eligible patients attending the OxVasc rapid-access TIA/stroke clinic underwent additional phenotyping (OxVasc Phenotyped Cohort), including TCD ultrasound and telemetric home blood pressure monitoring (HBPM); patients with presumed TIA or non-disabling stroke were eligible for inclusion in this study if they had no evidence of significant extra/intracranial stenosis of the anterior circulation (no stenosis or up to <50%), were willing and able to come back to clinic for one-month follow-up assessment and had a temporal bone window suitable for insonation.

During acute clinical assessment, brain and vascular imaging were obtained (Supplementary Methods). Demographic data, atherosclerotic risk factors including male sex, history of hypertension, diabetes mellitus, hypercholesterolemia, smoking habit (ex or current smoker), history of atrial fibrillation and ongoing medications were also recorded at initial face-to-face interview and cross-checked with primary care records.

Patients underwent TCD at two time-points: in the rapid-access TIA/stroke clinic and at the one-month follow-up visit. At both time points, TCD sonography (Doppler Box, Compumedics DWL, Singen, Germany) was performed by one of the three experienced operators (SM, MT, and LL), who were unaware of the patient’s clinical presentation, as detailed in the Supplementary Methods.

Patients carried on HBPM with a Bluetooth-enabled telemetric blood pressure monitor (IEM Stabil-o-Graph or A&D UA-767 BT) until at least the 1 month follow-up appointment, if tolerated (Supplementary Methods).

Secondary prevention treatment was started after initial assessment and included aspirin (300 mg loading and then 75mg daily), plus clopidogrel (300 mg loading dose and then 75 mg for 30 days) for high risk patients; atorvastatin (40–80 mg daily); antihypertensive treatment (unless systolic blood pressure was below 130 mm Hg on repeated measurement), according to a standardized protocol: a combination of perindopril arginine 5 mg and indapamide 1.25 mg followed by addition of amlodipine 5/10 mg, if necessary.^24^

### Statistical analysis

Analysis included all eligible patients recruited between 1 November 2011 and 30 November 2018 who underwent TCD ultrasound and had no evidence of ≥50%^25^ internal carotid artery (ICA) or MCA stenosis on vascular imaging. To reduce the risk of selection bias, the main analysis included patients recruited with what was considered initially to be a TIA, but in whom an alternative diagnosis was subsequently made on the basis of further investigation at follow-up,^24^ as well as patients with recurrent ischemic stroke between baseline and follow-up assessment.

Haemodynamic TCD measures of peak systolic velocity (PSV), end diastolic velocity (EDV), mean flow velocity (MFV), pulsatility index (PI) and resistance index (RI) were given as a mean/SD of the average of two measurements on each side at each time point.^24^ Measures of clinic systolic blood pressure (SBP) and diastolic blood pressure (DBP) were given as the mean/SD of two measurements taken before and after each TCD scan. Mean End-tidal CO_2_ (EtCo_2_) was given as the average of the readings recorded throughout the procedure at each time-point.

The difference between one-month follow-up and baseline measure for each variable was expressed as mean (mean one-month – mean baseline)/SD, with negative values reflecting a decrease between the two time-points.

Paired t test was used to compare blood pressure and haemodynamic parameters at baseline, at one-month follow-up and changes between the two time points in the analysis on the whole cohort, stratified by clinic and HBPM blood pressure and by age (<65 years of age, 65–79 or ‘elderly’ and ≥80 or ‘very old’ according to the 2018 European Society of Cardiology classification, ESC).^13^

To maximise precision of measurement of blood pressure changes, we also ran the analysis of hemodynamic changes in those patients in whom blood pressure reduction (with a target of <130/80) was guided by telemetric HBPM throughout the first month. ‘Hypertension’ at baseline was defined according to the ESC classification^13^ as office-based blood pressure ≥140/90 mmHg and HBPM-based ≥135/85 mmHg during the first 3 days of HBPM (Supplementary Methods).

Changes between baseline and follow-up of blood pressure and TCD parameters were analysed in the group of patients without antihypertensive treatment at baseline and follow-up, to explore the range of spontaneous variations of physiological parameters between the two time-points.

Sensitivity analyses were also done in patients presenting with SBP reduction ≥30 mmHg between baseline and follow-up TCD assessment; with SBP≥160 mmHg at baseline TCD assessment; with ≤7 days-interval since symptoms-onset; with an ischaemic lesion consistent with symptoms on magnetic resonance diffusion-weighted imagining (DWI).

Reliability of repeated measures of relevant variables at baseline and one-month follow-up was expressed as intra-class correlation coefficient with 95% confidence interval (CI).

To analyse the effect of changes between baseline and one-month follow-up in relevant TCD parameters change on long-term risk of subsequent recurrent stroke or death, Cox regression analysis was used, with age, sex and SBP at baseline as covariates.

All analyses were performed using SPSS version 26 and Stata version 16.1.

## Results

Of 821 eligible patients with an initial diagnosis of first-ever TIA/non-disabling stroke who had TCD in the rapid-access clinic **(**median time, IQR, between symptoms onset and TCD assessment: 3 days, 2–9**)**, 779 (94.9%) attended the one-month follow-up clinic for a second TCD assessment; 82 patients with **≥50%** intraor extracranial stenosis were excluded. Demographic and clinical characteristics of the remaining 697 patients included in the analysis are reported in Table 1. Fifty-two (7.5%) of these patients subsequently received a non-vascular diagnosis after completing investigations at one-month follow-up. Of 697 included patients, 335 (48%) were already on antihypertensive treatment at baseline assessment. Of the remaining 362 on no antihypertensive medications, 182 were prescribed at least one agent before the one-month follow-up clinic, such that 74% of patients were treated; antihypertensive medications were either started, added or increased in 67% of patients overall between their initial clinic assessment and one-month follow-up. The median (IQR) number of antihypertensive medications at baseline and one-month follow-up was 0 (1) and 2 (2), respectively. The most commonly used antihypertensive medications were calcium channel blockers (16.7% of patients at baseline and 41.2% at follow-up), ace-inhibitors/angiotensin receptor antagonists (33.7% of patients at baseline and 55.1% at follow-up) and diuretics (14.1% of patients at baseline and 36.5% at follow-up).

**Table 1.** Demographic and clinical characteristics of patients included in the analysis. Variables are expressed as N (%) unless stated otherwise. DWI = diffusion-weighted imaging; MRI = magnetic resonance imaging; mRS = modified Rankin Scale; TIA = transient ischaemic attack.

	Total *N* = 697
Age (mean/SD)	66.16/13.91
<65	52.15/9.18 (*N* = 279)
65–79	72.04/4.28 (*N* = 301)
≥80	84.43/3.71 (*N* = 117)
Male sex	414 (53.1%)
Time interval between symptoms onset and first assessment (median days, IQR)	3 (2–9)
Diagnosis	
TIA	433 (62.1%)
Stroke	212 (30.4%)
Mimics	52 (7.5%)
Risk factors	
History of hypertension	330 (47.3%)
History of smoking	354 (50.8%)
History of hyperlipidaemia	218 (31.3%)
History of atrial fibrillation	71 (10.2%)
History of diabetes	75 (10.8%)
DWI-positive on acute MRI imaging^∗^^[Table-fn table-fn1-23969873211039716]^	103 (18.2%)
Median mRS follow-up (IQR)	1 (0,2)

^*^patients with DWI positive lesion out of 565 patients who had MRI imaging in the acute phase.

**Figure 1.**
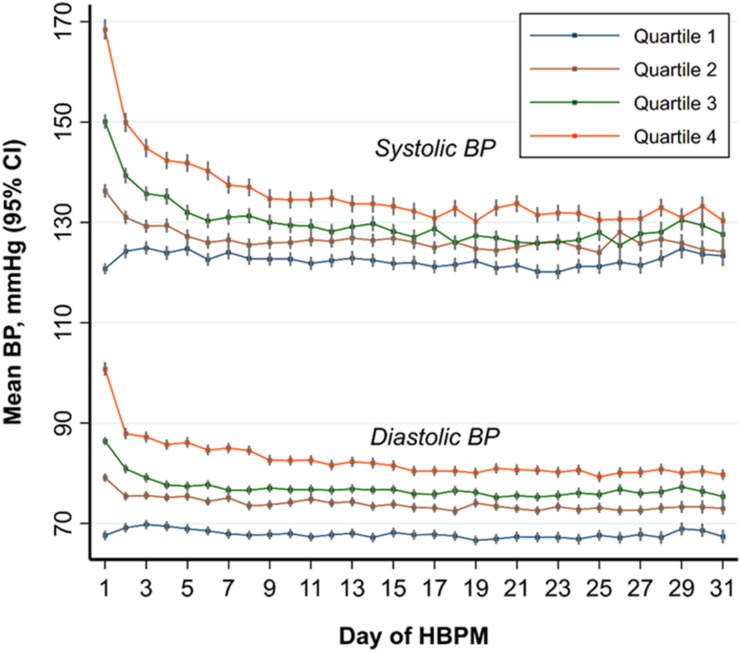
Systolic blood pressure and diastolic blood pressure on home blood pressure monitoring during the first month after clinical assessment, by quartiles. HBPM = home blood pressure monitoring.

Blood pressure (both clinic-based and HBPM) and haemodynamic parameters at baseline and follow-up and changes between the two time-points, are shown in Tables 2 and 3, respectively. Change over time in mean daily blood pressure on HBPM between baseline and one-month is shown in Figure 1. Overall, mean/SD baseline clinic SBP (145.0/21.3 mmHg) was reduced by an average of 11.3/19.9 mmHg (*p* < 0.0001) at 1 month (133.7/17.4 mmHg), mainly in patients who were hypertensive at baseline (SBP change = −19.0/19.2 mmHg, *p* < 0.001 vs −0.5/15.4, *p* = 0.62 in normotensives). Of all the TCD parameters, the only significant change observed between baseline and follow-up was an increase in EDV (0.77/7.26 cm/s, *p* = 0.005) and a decrease in RI (−0.005/0.051, *p* = 0.016), driven by hypertensive patients (mean/SD EDV change: 1.145/6.96 cm/s *p* = 0.001, RI change −0.007/0.06, *p* = 0.014 according to clinic blood pressure and 1.75/6.84 cm/s, *p* < 0.001 and −0.008/0.06, *p* = 0.049 for EDV and RI, respectively, according to HBPM), with larger changes in patients with blood pressure reduction between baseline and follow-up in the highest tertile (Table 3). In the normotensive group, changes were in the same direction, but smaller in absolute terms and non-statistically significant for either blood pressure measure modalities (Table 3). There were also significant increases between baseline and follow-up in MFV and PSV in the hypertensive subgroup (Supplementary Table 2). Changes in TCD parameters were unrelated to gender (Supplementary Table 3).

**Table 2.** Physiological variables (blood pressure and haemodynamic parameters) in the whole cohort at baseline, follow-up and difference between follow-up and baseline. SBP = clinic systolic blood pressure; DBP = clinic diastolic blood pressure; PSV = peak systolic velocity; EDV = end-diastolic velocity; MFV = mean flow velocity; PI = pulsatility index; RI = resistance index; EtCo_2_ = End-tidal Co_2_.

	Physiological variable	Baseline mean/SD	1 Month FU mean/SD	Difference mean/SD	*p*
**W**hole cohort *N* = 697	SBP mmHg	145.00/21.32	133.71/17.36	−11.29/19.90	<0.0001
	DBP mmHg	80.80/11.00	72.33/8.64	−8.28/10.65	<0.0001
	PSV cm/s	82.74/18.78	83.62/18.77	0.88/13.85	0.096
	EDV cm/s	33.52/10.28	34.29/10.64	0.77/7.26	0.005
	MFV cm/s	52.32/13.41	52.64/13.64	0.32/9.41	0.375
	PI	0.966/0.22	0.962/0.21	−0.003/0.143	0.476
	RI	0.597/0.074	0.592/0.076	−0.004/0.051	0.016
	EtCO_2_ kPa	5.14/0.67	5.39/0.81	0.25	<0.0001

**Table 3.** Changes in cerebral haemodynamic parameters during the first month after TIA/stroke by hypertension classification (office-based and home blood pressure monitoring-based), overall and in the top tertile of systolic blood pressure reduction. SBP = systolic blood pressure; EDV = end-diastolic velocity; RI = resistance index; TIA = transient ischaemic attack.

	Physiological variable	Overall				Top tertile of baseline to one-month SBP reduction			
		n	Baseline to one-month change	SD	p-value	N	Baseline to one-month change	SD	p-value
Assessment BP									
Whole cohort	Systolic BP		–11.3	19.9	**<0.001**		–32.2	13.7	**<0.001**
	EDV cm/s	697	0.770	7.26	**0.005**	234	1.302	6.90	**0.004**
	RI		–0.005	0.05	**0.016**		–0.013	0.06	**<0.001**
Hypertension (BP ≥ 140/90 mmHg)	Systolic BP		–19.0	19.2	**<0.001**		–33.3	14.0	**<0.001**
	EDV cm/s	407	1.145	6.96	**0.001**	208	1.374	6.89	**0.005**
	RI		–0.007	0.06	**0.014**		–0.014	0.06	**0.001**
Normotension (BP < 140/90 mmHg)	Systolic BP		–0.5	15.4	0.621		–23.2	4.9	**<0.001**
	EDV cm/s	289	0.237	7.65	0.601	26	0.721	7.12	0.610
	RI		–0.002	0.04	0.541		–0.010	0.04	0.225
HBPM									
Subset with HBPM	Systolic BP		–7.6	11.9	**<0.001**		–20.8	8.4	**<0.001**
	EDV cm/s	457	1.036	6.77	**0.001**	146	2.28	6.79	**<0.001**
	RI		–0.005	0.05	**0.038**		–0.01	0.06	**0.013**
Hypertension (BP ≥ 135/85 mmHg)	Systolic BP		–13.2	12.2	**<0.001**		–22.0	8.9	**<0.001**
	EDV cm/s	226	1.75	6.84	**<0.001**	116	2.707	6.90	**<0.001**
	RI		–0.008	0.06	**0.049**		–0.011	0.06	**0.045**
Normotension (BP < 135/85 mmHg)	Systolic BP		–2.2	8.6	**<0.001**		–16.3	3.5	**<0.001**
	EDV cm/s	231	0.084	7.44	0.857	30	0.642	6.16	0.573
	RI		–0.003	0.05	0.379		–0.014	0.04	0.090

Changes in EDV and RI were consistent across age groups (EDV change–p_trend_ = 0.357; RI change–p_trend_ = 0.225), including 117 patients ≥80 (mean/SD age 84.43/3.71), where haemodynamic changes similar to those in the younger age groups were observed, despite somewhat greater blood pressure decrease between baseline and one-month follow-up (Table 4 and Supplementary Table 4).

**Table 4.** Age-specific analysis of blood pressure and hemodynamic parameters change between baseline and one-month follow-up. SBP = clinic systolic blood pressure; EDV = end-diastolic velocity; RI = resistance index.

	Age groups (years of age)			
Physiological variables	**<65** (*N* = 279)	**65–79** (*N* = 301)	**≥80** (*N* = 117)	*Difference p*
SBP change mmHg (Mean/SD)	−10.94/17.24	−10.66/19.99	−13.72/25.00	0.347
EDV change cm/s (Mean/SD)	0.29/8.34	1.10/6.84	1.09/5.20	0.357
RI change (Mean/SD)	−0.003/0.052	−0.008/0.05	−0.00002/0.053	0.225

Intra-class correlation coefficients for EDV and RI at baseline and follow-up were 0.862, 95%CI 0.839–0.881 and 0.867, 95%CI 0.846–0.885, respectively.

Mean/SD EtCO_2_ significantly increased between baseline (5.14/0.67 KPa) and follow-up (5.39/0.81 KPa, mean/SD change = 0.25 KPa, *p* < 0.0001). The EtCO2 change did not differ across gender (mean/SD EtCO2 change = 0.25/0.61 KPa in men vs 0.24/0.63 in women *p* = 0.863) and age (mean/SD EtCO2 change = 0.26/0.58 vs 0.26/0.67 vs 0.21/0.56 for <65, 65–79 and ≥80 years of age, respectively, *p* = 0.832) groups.

Between the first and the second assessment, the group of 180 patients on no antihypertensive treatment at baseline and follow-up displayed EDV changes ranging between −19.75 and 20 cm/s. In the 517 patients on antihypertensive medication (either already on medication or started at baseline), EDV changes ranged between −23.50 and 37.50 cm/s. Only two of these patients had an EDV decrease between baseline and follow-up below −19.75 cm/s (−23.50 and −20.75 cm/s), both with PSV at baseline suggestive of possible MCA stenosis, which resolved on subsequent assessment and was not demonstrated at the time of vascular imaging. These two patients did not have any excess adverse events on long-term follow-up. In the sub-group with more intensive SBP reduction (≥30 mmHg), EDV changes ranged between −17.25 and 24 cm/s p-values in bold are statistically significant (p < 0.05).

In the other sensitivity analyses (Supplementary Table 1), results were consistent when excluding patients with an ultimate non-vascular diagnosis at one-month follow-up and after excluding three patients with recurrent ischemic stroke between baseline and follow-up assessment. Haemodynamic changes were also consistent in patients with SBP≥160 mmHg at baseline, those with symptoms onset 7 days before baseline assessment or less (median time-interval between the index event and baseline assessment, days/IQR = 2/1-4) and those with an acute ischaemic lesion consistent with symptoms on magnetic-resonance DWI. The largest absolute changes in haemodynamic parameters were observed in the 100 patients with mean clinic SBP decrease of 30 mmHg or more, with mean/SD increase in EDV of 2.49/7.47 cm/s (*p* = 0.001) and decrease in RI of −0.024/0.063 (*p* < 0.0001) (Supplementary Table 1).

Increase in EDV at one-month follow-up was not associated with a higher risk of recurrent stroke or death on long-term (mean/SD = 4.31/1.84 years) follow-up (age-adjusted Hazard Ratio 1.04, 95% CI 0.67–1.61, *p* = 0.873).

## Discussion

Our study showed no evidence of detrimental effects of intensive blood pressure lowering soon after TIA/non-disabling stroke on TCD blood flow velocities, particularly in elderly (65–79 years) and very old (≥80 years) patients. In fact, a significant increase in TCD EDV was consistent across all age groups, with no evidence that older ages are at disproportionately higher risk of reduced velocities with intensive blood pressure reduction early after TIA/non-disabling stroke. This is a clinically important finding given that many elderly patients are treated in rapid-access TIA clinics and that there is some uncertainty around timing and targets for blood pressure reduction in the context of secondary prevention of stroke in this age group.

As the autoregulatory response to blood pressure variations takes place predominantly in small parenchymal vessels distal to the proximal MCA,^26^ the EDV increase and RI decrease observed in our study after blood pressure reduction suggest, in presence of constant proximal MCA calibre, there is a decrease in distal vascular resistance vessels. EDV increase has been shown to reflect increased perfusion in the clinical setting of pharmacological cerebral reperfusion after stroke, with even a small increase in early post-recanalisation EDV being associated with significant neurological and functional improvement, suggesting that EDV is a clinically relevant marker of cerebral perfusion.^27^

**Figure 2.**
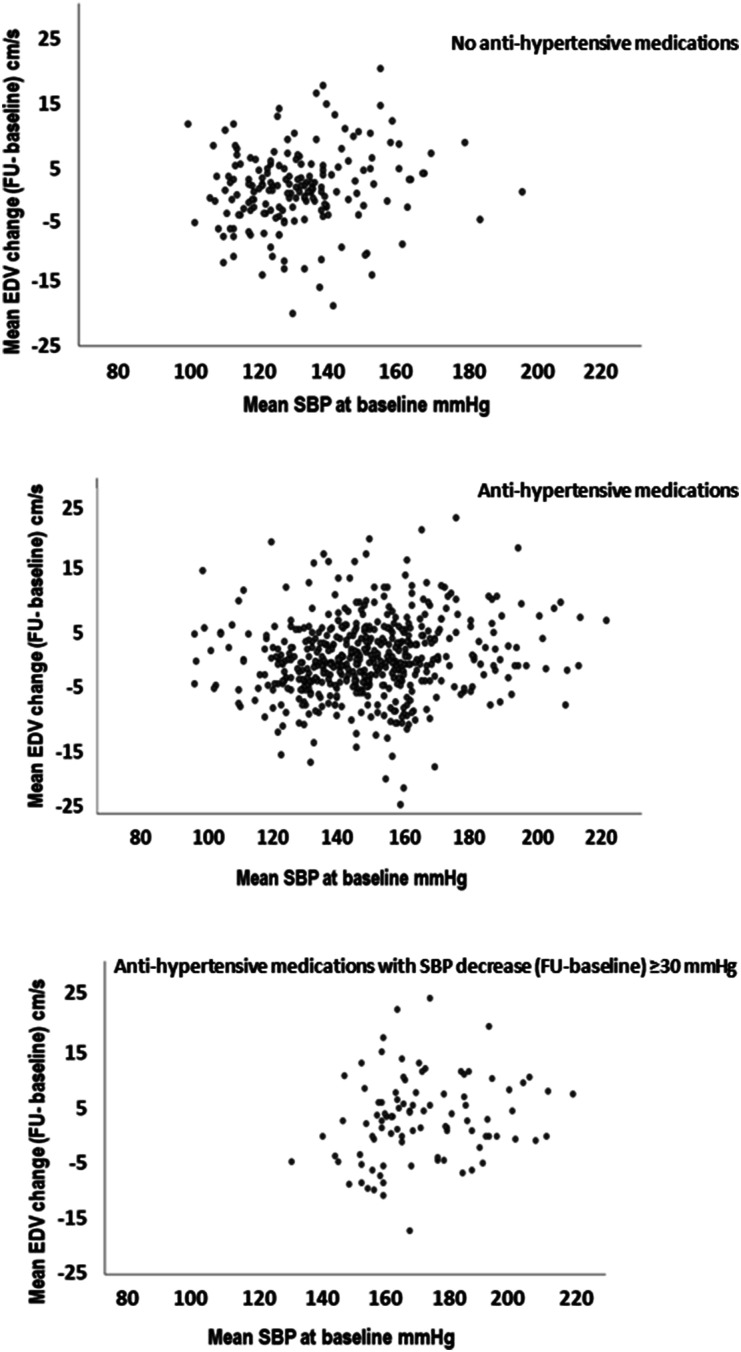
Correlation between mean systolic blood pressure at baseline and mean EDV change between baseline and follow-up in patients on no antihypertensive medication at baseline and follow-up; in patients on antihypertensive medication; and in patients on antihypertensive medication with blood pressure reduction between baseline and follow-up >30 mmHg. SBP = systolic blood pressure; EDV = end-diastolic velocity; FU = follow-up.

Interestingly, absolute changes in EDV and RI were largest in patients with hypertension at baseline and in those with SBP reduction of 30 mmHg or more, with no evidence of disproportionate EDV decrease in any of these patients (Figure 2). Reduction in blood pressure was seen on both clinic measurements and on detailed HBPM and was not simply a statistical phenomenon of regression to the mean based on limited measurement.

The direction and magnitude of haemodynamic changes were un-altered in our sensitivity analyses, including in patients who might be at higher risk of altered cerebral autoregulation due to cerebral infarction,^28^ such as patients with more recent symptoms-onset (the median time interval between the index event and baseline assessment was days/IQR=2/1-4), or with DWI-positive lesions on MRI imaging.

The observed increase in EtCO_2_, albeit small in absolute terms, might have contributed to the increase in EDV between baseline and follow-up, and could partly reflect a state of anxiety – with relative hypocapnia – in patients in the acute setting, which was attenuated at 1 month follow-up.^24^ Indeed, there is a positive linear association between TCD velocities and partial arterial CO_2_ pressure, which is made steeper by anxiety.^29^ However, the fact that TCD changes were only seen in hypertensive patients makes it unlikely that that they were entirely due to anxiety-related changes in EtCO_2_.

This study has several strengths. It is the first large study on cerebral haemodynamic effects of blood pressure lowering in symptomatic patients soon after TIA/non-disabling stroke, allowing detection of small physiological changes that would otherwise go overlooked. Importantly, we included a large proportion (about two-thirds) of patients ≥65 years of age, 117 of whom ≥80, who were followed up 4 weeks after the first assessment, when autoregulation is more likely to have settled after a vascular event. It was a pragmatic, real-world study, conducted in an every-day clinical setting, therefore providing more clinically useful information to clinicians than physiologically sophisticated studies removed from routine clinical conditions.

However, our study also has some weakness. Firstly, we used TCD sonography, which provides a measure of blood flow velocity in the basal cerebral arteries rather than of blood flow.^30^ However, TCD is an accurate and easily accessible method for functional studies of cerebral haemodynamics, ^26,31^ and a strong correlation between blood flow volume and TCD blood flow velocity in the proximal MCA has been demonstrated.^32^ Although it could be argued that pharmacologically induced blood pressure reductions could result in changes of MCA diameter,^32^ experimental studies suggest that any such effects are negligible in the proximal MCA, with changes confined to the smaller distal vessels.^33^ Moreover, minor changes in proximal MCA diameter would be unlikely to cause unacceptable discrepancy between velocity and flow in most cases.^32^ Furthermore, other imaging methods, including Xe inhalation method^19^ and single photon emission computed tomography^20^ arterial spin labelling magnetic resonance imaging,^4^ are less practical in large studies and more likely to lead to exclusion of older and frail patients. Our findings highlight the potential of TCD EDV and RI as potential surrogate markers of cerebral blood flow in future studies and trials targeting cerebral perfusion.^27^ Secondly, some antihypertensive medications have direct and specific effects on cerebral blood flow,^34,35^ but we were unable to assess this, as the majority of our patients were treated with multiple agents. Thirdly, although the magnitude and direction of EDV changes between baseline and follow-up was consistent across all sensitivity analyses, they represent mean changes; we have however shown that the range of changes in the group of patients receiving pharmacological blood pressure lowering was similar to that of patients whose blood pressure was not pharmacologically reduced, with a shift towards higher value of EDV increase rather than towards EDV decrease. Lastly, these results cannot be generalised to patients with significant intra/extracranial stenosis, who were excluded from the present analysis. Further studies are needed in this group of patients**.**

## Conclusions

In this study on patients without arterial stenosis, there was no suggestion on TCD sonography of decreased blood flow velocities associated to blood pressure lowering soon after TIA and non-disabling stroke, irrespective of age, including elderly (65–79 years) and very old (≥80 years) patients. Rather, the observed EDV increase and RI decrease suggest reduction in distal vascular resistance.

## Supplemental Material

sj-pdf-1-eso-10.1177_23969873211039716 – Supplemental Material for Age-specific cerebral haemodynamic effects of early blood pressure lowering after transient ischaemic attack and non-disabling stroke Click here for additional data file. Supplemental Material, sj-pdf-1-eso-10.1177_23969873211039716 for Age-specific cerebral haemodynamic effects of early blood pressure lowering after transient ischaemic attack and non-disabling stroke by Sara Mazzucco, Linxin Li, Iain J McGurgan, Maria A Tuna, Nicoletta Brunelli, Lucy E Binney, Peter M Rothwell and on behalf of the Oxford Vascular Study Phenotyped cohort in European Stroke Journal
