# Supplementary material for: Age-specific cerebral haemodynamic effects of early blood pressure lowering after transient ischaemic attack and non-disabling stroke
Source: Eur Stroke J. 2021 Sep 4;6(3):245–53. doi: 10.1177/23969873211039716 (PMC8564162; doi:10.1177/23969873211039716)
Supplement: sj-pdf-1-eso-10.1177_23969873211039716 – Supplemental Material for Age-specific cerebral haemodynamic effects of early blood pressure lowering after transient ischaemic attack and non-disabling stroke [file sj-pdf-1-eso-10.1177_23969873211039716.pdf]

SUPPLEMENTAL MATERIAL

Age-specific cerebral haemodynamic effects of early blood pressure lowering after TIA and non-disabling stroke

Sara Mazzucco, MD PhD,[1] Linxin Li,[1] MD DPhil, Iain J McGurgan, MD,[1] Maria Assuncao Tuna,[1] MD DPhil, Nicoletta Brunelli, MD,[2] Lucy E Binney, MD,[1] Peter M Rothwell, MD,[1] PhD, FmedSci, on behalf of the Oxford Vascular Study Phenotyped cohort

[1] Wolfson Centre for Prevention of Stroke and Dementia, Nuffield Department of Clinical Neurosciences, University of Oxford

[2] Campus Bio-Medico University of Rome, Rome, Italy

Supplementary methods:

1. **OXVASC methodology**

Study population

The Oxford Vascular Study (OXVASC) is a prospective, population-based cohort study of all incident acute vascular events in all territories (transient ischaemic attack, stroke, acute coronary and peripheral vascular events).[1,2] During the period of the current substudy, the OXVASC study population consisted of all 92,728 individuals, irrespective of age, registered with 100 general practitioners (GPs) in nine general practices in Oxfordshire, UK. In the UK, general practices provide primary health care for registered individuals and hold a lifelong record of all medical consultations (from the National Health Service [NHS] and private health care), and details of treatments, blood pressure, and investigations. In Oxfordshire, an estimated 97% of the true residential population is registered with a general practice, with most non-registered individuals being young students. All participating practices held accurate age-sex patient registers, and allowed regular searches of their computerised diagnostic coding systems. The practices had all collaborated on a previous population-based study, for which they were originally selected to be representative of the urban and rural mix and the deprivation range of Oxfordshire as a whole.[3] Based on the index of multiple deprivation (IMD), the population was less deprived than the rest of England, but had a broad range of deprivation.

The OXVASC population is 94% white people, 3% Asian, 2% Chinese, and 1% Afro-Caribbean.[4] The proportion of whites is similar to that of the UK as a whole (88% white) and to many other western countries (Australia - 90%; France - 91%; Germany - 93.9%).

Case ascertainment

After a 3-month pilot study, the study started on April 1, 2002, and is ongoing. Ascertainment combined prospective daily searches for acute events (hot pursuit) and retrospective searches of hospital-care and primary-care administrative and diagnostic coding data (cold pursuit).

Hot pursuit was based on:

1. A daily (weekdays only), urgent open-access “TIA clinic” to which participating general practitioners (GPs) and the local accident and emergency department (A&E) send all individuals with suspected TIA or stroke whom they would not normally admit to hospital, with alternative on-call review provision at weekends. Patients too frail to attend are assessed at their residence by a study nurse or doctor.
2. Daily searches and case note review of admissions to the Emergency Assessment Unit, Medical Short Stay Unit, Coronary Care Unit and Cardiothoracic Critical Care Unit, Cardiology, Cardiothoracic, and Vascular Surgery wards, Acute Stroke Unit, Neurology ward and all other general wards when indicated.
3. Daily searches of the local A&E and eye hospital attendance registers.
4. Daily identification via the Bereavement Office of patients dead on arrival at hospital or who died soon after.
5. Daily searches of lists of all patients from the study population in whom a troponin-I level had been requested.
6. Daily assessment of all patients undergoing diagnostic coronary, carotid and peripheral angiography, angioplasty, stenting or vascular surgical procedures in any territory to identify both total burden of vascular invention and any potential missed prior acute events.

Cold pursuit procedures were:

1. Frequent visits to the study practices and monthly searches of practice diagnostic codes.
2. Monthly practice-specific list of all patients admitted to all acute and community NHS hospitals.
3. Monthly listings of all referrals for brain or carotid imaging studies performed in local hospitals.
4. Monthly reviews of all death certificates and coroners reports to review out-of-hospital deaths.
5. Practice-specific listings of all ICD-10 death codes from the local Department of Public Health.

Patients found on GP practice searches who have an event whilst temporarily out of Oxfordshire are included, but visitors who were not registered with one of the study practices are excluded. A study clinician assessed patients as soon as possible after the event in the hospital or at home. Informed consent was sought, if possible, or assent was obtained from a relative. Data are collected using event-specific forms, for TIA and stroke, acute coronary syndrome or acute peripheral vascular events. Standardised clinical history and cardiovascular examination are recorded. Information recorded from the patient, their hospital records and their general practice records includes details of the clinical event, medication, past medical history, all investigations relevant to their admission (including blood results, electrocardiography, brain imaging and vascular imaging-duplex ultrasonography, CT-angiography, MR-angiography or DSA) and all interventions occurring subsequent to the event.

If a patient died before assessment, we obtained an eyewitness account of the clinical event and reviewed any relevant records. If death occurred outside the hospital or before investigation, the autopsy result was reviewed. Clinical details are sought from primary care physicians or other clinicians on all deaths of possible vascular aetiology.

All surviving TIA and stroke patients are followed-up face-to-face at 1, 6, 12, 60 and 120 months after the initial event by a research nurse or physician and all recurrent vascular events were recorded together with the relevant clinical details and investigations. If face-to-face follow up is not possible, telephone follow-up is performed or enabled via the general practitioner. All recurrent vascular events that presented to medical attention would also be identified acutely by ongoing daily case ascertainment within OXVASC. If a recurrent vascular event was suspected at a follow-up visit or referred by the GPs to clinic or admitted, the patient was re-assessed and investigated by a study physician.

Definitions of events

Although new definitions for stroke and TIA have been suggested recently,[4,5] in order to enable comparison with previous studies, the classic definitions of TIA and stroke are used throughout.[6] A stroke is defined as rapidly developing clinical symptoms and/or signs of focal, and at time global (applied to patients in deep coma and to those with subarachnoid haemorrhage), loss of brain function, with symptoms lasting more than 24 hours or leading to death, with no apparent cause other than that of vascular origin.[6] A TIA is an acute loss of focal brain or monocular function with symptoms lasting less than 24 hours and which is thought to be caused by inadequate cerebral or ocular blood supply as a result of arterial thrombosis, low flow or embolism associated with arterial, cardiac or haematological disease.[4] All diagnoses were reviewed by a senior neurologist (PMR). With the high rate (97%) of imaging or autopsy in OXVASC, strokes of unknown type were coded as ischaemic.

2. Brain and vascular imaging

During the acute clinical assessment, brain and vascular imaging are obtained, either 3T magnetic resonance imaging (MRI) with time-of-flight magnetic resonance angiography (MRA) of the intracranial vessels and a contrast-enhanced MRA of the large neck arteries, or brain computed tomography (CT) with contrast-enhanced CT angiography or Duplex ultrasound if MRI is contraindicated.[7]

3. Transcranial Doppler and capnometry protocol

Middle cerebral artery blood flow velocity was recorded with a handheld 2 MHz probe through temporal bone window at the depth that provided the best signal, usually 50 mm. Transcranial Doppler (TCD) examination was conducted in a quiet room, with the patient lying comfortably on a couch, having a lying blood pressure measure taken before and after the scan. Each session was stored in the hard disk of the TCD device for subsequent off-line analysis.[8]

End-tidal CO_2 was monitored via nasal cannulae (Capnocheck Plus; Smith Medical) throughout the procedure at each time point.

4. Home blood pressure monitoring

Patients were fitted with a Bluetooth-enabled telemetric blood pressure monitor (IEM Stabil-o-Graph or A&D UA-767 BT) in clinic on the day of assessment (or at the earliest opportunity). After appropriate training, they were instructed to perform sets of three home readings in the non-dominant arm, or the arm with the higher reading (if mean blood pressure differed by >20mmHg between arms), three times daily (on waking, mid-morning and before sleep). Measurements were transmitted by Bluetooth radio to a mobile phone or Raspberry Pi microcomputer hub for secure transmission to a server, hosting a password-protected website for review and download of readings (t+ Medical, Abingdon, UK), and were assessed daily by the OxVasc team. Patients continued home monitoring until at least the one month follow-up appointment, if tolerated.[9]

Home blood pressure monitoring (HBPM) readings for each participant were downloaded from the encrypted website and manually assessed to remove those with erroneous measures or incorrect date/time stamps (n=23, <0.1%). Recordings with SBP <50 mmHg (n=1), DBP >140 mmHg if pulse pressure <40 mmHg (n=2), and any pulse pressure <10 mmHg (n=2) were excluded. The first three days of HBPM were used for diagnosis of hypertension (BP ≥135/85mmHg). If the first HBPM reading coincided with the date and time of the baseline clinic reading, this was excluded, as some patients were instructed to take a test reading as part of their instruction in clinic on how to use the HBPM kit.

References

1. Rothwell PM, Coull AJ, Giles MF, et al. Change in stroke incidence, mortality, case-fatality, severity, and risk factors in Oxfordshire, UK from 1981 to 2004 (Oxford Vascular Study). Lancet 2004; 363: 1925–33.
2. Rothwell PM, Coull AJ, Silver LE, Fairhead JF, Giles MF, Lovelock CE, Redgrave JNE, Bull LM, Welch SJV, Cuthbertson FC, Binney LE, Gutnikov SA, Anslow P, Banning AP, Mant D, Mehta Z for the Oxford Vascular Study. Population-based study of event-rate, incidence, case fatality and mortality for all acute vascular events in all arterial territories (Oxford Vascular Study). Lancet 2005; 366: 1773-83.

3. Bamford J, Sandercock P, Dennis M, Burn J, Warlow C. A prospective study of acute cerebrovascular disease in the community: the Oxfordshire Community Stroke Project--1981-86. 2. Incidence, case fatality rates and overall outcome at one year of cerebral infarction, primary intracerebral and subarachnoid haemorrhage. *J Neurol Neurosurg Psychiatry* 1990;53:16-22.
4. Easton JD, Saver JL, Albers GW, et al. Definition and evaluation of transient ischemic attack: a scientific statement for healthcare professionals from the American Heart Association/American Stroke Association Stroke Council; Council on Cardiovascular Surgery and Anesthesia; Council on Cardiovascular Radiology and Intervention; Council on Cardiovascular Nursing; and the Interdisciplinary Council on Peripheral Vascular Disease. The American Academy of Neurology affirms the value of this statement as an educational tool for neurologists. *Stroke* 2009;40:2276-2293.
5. Sacco RL, Kasner SE, Broderick JP, et al. An updated definition of stroke for the 21st century: a statement for healthcare professionals from the American Heart Association/American Stroke Association. *Stroke* 2013;44:2064-2089.
6. Hatano S. Experience from a multicentre stroke register: a preliminary report. *Bulletin of the World Health Organization* 1976;54:541-553.
7. Li L, Yiin GS, Geraghty OC, et al. Incidence, outcome, risk factors, and long-term prognosis of cryptogenic transient ischaemic attack and ischaemic stroke: a population-based study. *Lancet Neurol.* 2015;14(9):903–13.
8. Mazzucco S, Li L, Tuna MA, Pendlebury ST, Wharton R, PM Rothwell. Hemodynamic correlates of transient cognitive impairment after transient ischemic attack and minor stroke: A transcranial Doppler study, and on behalf of the Oxford Vascular Study. Int J Stroke 2016; 11:978–986.
9. Webb AJS, Wilson M, Lovett N, Paul N, Fischer U, Rothwell PM. Response of day-to-day home blood pressure variability by antihypertensive drug class after TIA or non-disabling stroke. Stroke 2014;45:2967–2973.

Supplementary Table 1: Sensitivity analyses

Patients N	Physiological variable	Baseline Mean/SD	1 Month FU Mean /SD	Difference Mean/SD	p
**Systolic blood pressure decrease of 30 mmHg or more**					
	**SBP mmHg**	173.00/18.36	128.59/14.59	-44.37/12.85	**<0.0001**
**100**	**EDV cm/s**	30.18/9.49	32.68/9.91	2.49/7.47	**0.001**
	**RI**	0.619/0.073	0.595/0.076	-0.024/0.063	**<0.0001**
**Baseline systolic blood pressure ≥160 mmHg**					
	**SBP mmHg**	173.76/13.94	143.91/18.21	-29.85/20.41	**<0.0001**
**160**	**EDV cm/s**	29.22/9.46	31.08/9.56	1.59/7.16	**0.003**
	**RI**	0.640/0.066	0.628/0.077	-0.011/0.061	**0.019**
**Symptoms onset ≤7 days before baseline assessment**					
	**SBP mmHg**	144.91/21.51	133.50/17.17	-11.41/20.10	**<0.0001**
**492**	**EDV cm/s**	33.16/10.33	34.32/10.89	1.16/7.55	**0.001**
	**RI**	0.600/0.076	0.594/0.078	-0.005/0.051	**0.018**
**DWI positive lesion present on magnetic resonance at baseline**					
	**SBP mmHg**	149.44/22.89	134.85/16.09	-14.59/20.32	**<0.0001**
**103**	**EDV cm/s**	32.06/10.56	33.96/10.68	1.90/6.40	**0.004**
	**RI**	0.599/0.081	0.594/0.075	-0.005/0.049	0.292
**Final diagnosis of TIA/stroke**					
	**SBP mmHg**	145.26/21.16	133.54/17.14	-11.71/20.04	**<0.0001**
**644**	**EDV cm/sec**	33.35/10.29	34.07/10.63	0.72/7.43	**0.015**
	**RI**	0.600/0.074	0.593/0.077	-0.004/0.052	**0.034**
**With no recurrent events between baseline and follow-up**					
	**SBP mmHg**	145.01/21.34	133.71/17.37	-11.29/19.93	**<0.0001**
**694**	**EDV cm/s**	33.50/10.29	34.28/10.64	0.77/7.61	**0.005**
	**RI**	0.597/0.074	0.593/0.076	-0.005/0.051	**0.016**

Physiological variables (clinic blood pressure and haemodynamic parameters) at baseline, one-month follow-up and difference between baseline and follow -up for the sensitivity analyses. SBP= clinic systolic blood pressure; EDV= end-diastolic velocity; RI= resistance index, ICA= internal carotid artery; MCA= middle cerebral artery

Supplementary Table 2. Changes in TCD parameters between baseline and follow-up stratified by blood pressure measures in the whole cohort and in patients with HBPM. SBP= clinic systolic blood pressure; PSV= peak systolic velocity; EDV= end-diastolic velocity; MFV= mean flow velocity; PI= pulsatility index; RI= resistance index.

		Overall				Top tertile of baseline to one-month SBP reduction			
	**Physiological variable**	**n**	**Baseline to one-month change**	**SD**	**p-value**	**n**	**Baseline to one-month change**	**SD**	**p-value**
**Assessment BP**									
Whole cohort	**Systolic BP**	697	-11.3	19.9	**<0.001**	234	-32.2	13.7	**<0.001**
	**EDV cm/s**		0.770	7.26	**0.005**		1.302	6.90	**0.004**
	**MFV cm/s**		0.317	9.41	0.375		0.242	9.24	0.689
	**PSV cm/s**		0.877	13.8	0.096		0.903	13.91	0.322
	**PI**		-0.004	0.14	0.476		-0.018	0.16	0.093
	**RI**		-0.005	0.05	**0.016**		-0.013	0.06	**<0.001**
Hypertension (BP ≥ 140/90 mmHg)	**Systolic BP**	407	-19.0	19.2	**<0.001**	208	-33.3	14.0	**<0.001**
	**EDV cm/s**		1.145	6.96	**0.001**		1.374	6.89	**0.005**
	**MFV cm/s**		0.479	9.55	0.313		0.266	9.28	0.679
	**PSV cm/s**		1.551	14.64	**0.034**		1.046	14.04	0.285
	**PI**		-0.004	0.16	0.589		-0.018	0.17	0.127
	**RI**		-0.007	0.06	**0.014**		-0.014	0.06	**0.001**
Normotension (BP < 140/90 mmHg)	**Systolic BP**	289	-0.5	15.4	0.621	26	-23.2	4.9	**<0.001**
	**EDV cm/s**		0.237	7.65	0.601		0.721	7.12	0.610
	**MFV cm/s**		0.088	9.23	0.871		0.048	9.13	0.979
	**PSV cm/s**		-0.079	12.59	0.915		-0.231	13	0.929
	**PI**		-0.003	0.12	0.633		-0.173	0.09	0.351
	**RI**		-0.002	0.04	0.541		-0.010	0.04	0.225
**HBPM**									
Subset with HBPM	**Systolic BP**	457	-7.6	11.9	**<0.001**	146	-20.8	8.4	**<0.001**
	**EDV cm/s**		1.036	6.77	**0.001**		2.28	6.79	**<0.001**
	**MFV cm/s**		0.714	9.18	0.095		1.95	8.72	**0.006**
	**PSV cm/s**		1.773	13.98	**0.007**		3.16	13.51	**0.005**
	**PI**		-0.004	0.14	0.537		-0.02	0.15	0.054
	**RI**		-0.005	0.05	**0.038**		-0.01	0.06	**0.013**
Hypertension (BP ≥ 135/85 mmHg)	**Systolic BP**	226	-13.2	12.2	**<0.001**	116	-22.0	8.9	**<0.001**
	**EDV cm/s**		1.75	6.84	**<0.001**		2.707	6.90	**<0.001**
	**MFV cm/s**		1.336	8.96	**0.025**		2.536	13.31	**0.001**
	**PSV cm/s**		2.68	13.86	**0.004**		4.059	13.31	**0.001**
	**PI**		-0.011	0.16	0.273		-0.025	0.16	0.077
	**RI**		-0.008	0.06	**0.049**		-0.011	0.06	**0.045**
Normotension (BP < 135/85 mmHg)	**Systolic BP**	231	-2.2	8.6	**<0.001**	30	-16.3	3.5	**<0.001**
	**EDV cm/s**		0.084	7.44	0.857		0.642	6.16	0.573
	**MFV cm/s**		0.096	9.37	0.877		-0.464	8.96	0.779
	**PSV cm/s**		0.889	14.07	0.339		-0.475	13.93	0.853
	**PI**		0.004	0.13	0.646		-0.016	0.12	0.461
	**RI**		-0.003	0.05	0.379		-0.014	0.04	0.090

Supplementary Table 3: Transcranial Doppler parameters changes between baseline and follow-up and by gender. PSV= peak systolic velocity; EDV= end-diastolic velocity; MFV= mean flow velocity; PI= pulsatility index; RI= resistance index.

	**Men**	**Women**	**p**
**Mean/SD PSV change, cm/s**	1.44/13.36	0.26/14.36	0.266
**Mean/SD EDV change, cm/s**	0.99/7.48	0.53/7.01	0.406
**Mean/SD MFV change, cm/s**	0.83/9.15	-0.24/9.67	0.131
**Mean/SD PI change**	-0.006/0.15	-0.002/0.13	0.733
**Mean/SD RI change**	-0.004/0.05	-0.005/0.05	0.755

Supplementary Table 4: Transcranial Doppler parameters changes between baseline and follow-up by age group. PSV= peak systolic velocity; EDV= end-diastolic velocity; MFV= mean flow velocity; PI= pulsatility index; RI= resistance index.

	**<65 years**	**65-79 years**	**≥80 years**	**p**
**Mean/SD PSV change, cm/s**	-0.25/13.72	1.21/14.58	2.76/11.93	0.127
**Mean/SD EDV change, cm/s**	0.29/8.34	1.10/6.84	1.09/5.20	0.357
**Mean/SD MFV change, cm/s**	-0.14/9.98	0.40/9.61	1.19/7.28	0.432
**Mean/SD PI change**	-0.002/0.13	-0.01/0.14	0.006/0.18	0.548
**Mean/SD RI change**	-0.003/0.05	-0.008/0.05	0.00002/0.53	0.225
